# Evaluation of inter- and intra-operator reliability of manual segmentation of femoral metastatic lesions

**DOI:** 10.1007/s11548-021-02450-w

**Published:** 2021-07-15

**Authors:** Ali Ataei, Florieke Eggermont, Milan Baars, Yvette van der Linden, Jacky de Rooy, Nico Verdonschot, Esther Tanck

**Affiliations:** 1 Orthopaedic Research Lab, Radboud University Medical Center, Radboud Institute for Health Sciences, P.O. Box 9101, 6500 HB Nijmegen, The Netherlands; 2 Department of Clinical Oncology, Leiden University Medical Center, Leiden, The Netherlands; 3 Department of Radiology, Radboud University Medical Center, Nijmegen, The Netherlands; 4 Laboratory for Biomechanical Engineering, University of Twente, Enschede, The Netherlands

**Keywords:** Bone metastasis, Manual segmentation, Reliability, Femoral lesions

## Abstract

**Purpose:**

Accurate identification of metastatic lesions is important for improvement in biomechanical models that calculate the fracture risk of metastatic bones. The aim of this study was therefore to assess the inter- and intra-operator reliability of manual segmentation of femoral metastatic lesions.

**Methods:**

CT scans of 54 metastatic femurs (19 osteolytic, 17 osteoblastic, and 18 mixed) were segmented two times by two operators. Dice coefficients (DCs) were calculated adopting the quantification that a DC˃0.7 indicates good reliability.

**Results:**

Generally, rather poor inter- and intra-operator reliability of lesion segmentation were found. Inter-operator DCs were 0.54 (± 0.28) and 0.50 (± 0.32) for the first and second segmentations, respectively, whereas intra-operator DCs were 0.56 (± 0.28) for operator I and 0.71 (± 0.23) for operator II. Larger lesions scored significantly higher DCs in comparison with smaller lesions. Of the femurs with larger mean segmentation volumes, 83% and 93% were segmented with good inter- and intra-operator DCs (> 0.7), respectively. There was no difference between the mean DCs of osteolytic, osteoblastic, and mixed lesions.

**Conclusion:**

Manual segmentation of femoral bone metastases is very challenging and resulted in unsatisfactory mean reliability values. There is a need for development of a segmentation protocol to reduce the inter- and intra-operator segmentation variation as the first step and use of computer-assisted segmentation tools as a second step as this study shows that manual segmentation of femoral metastatic lesions is highly challenging.

**Supplementary Information:**

The online version contains supplementary material available at 10.1007/s11548-021-02450-w.

## Introduction

**Fig. 1:**
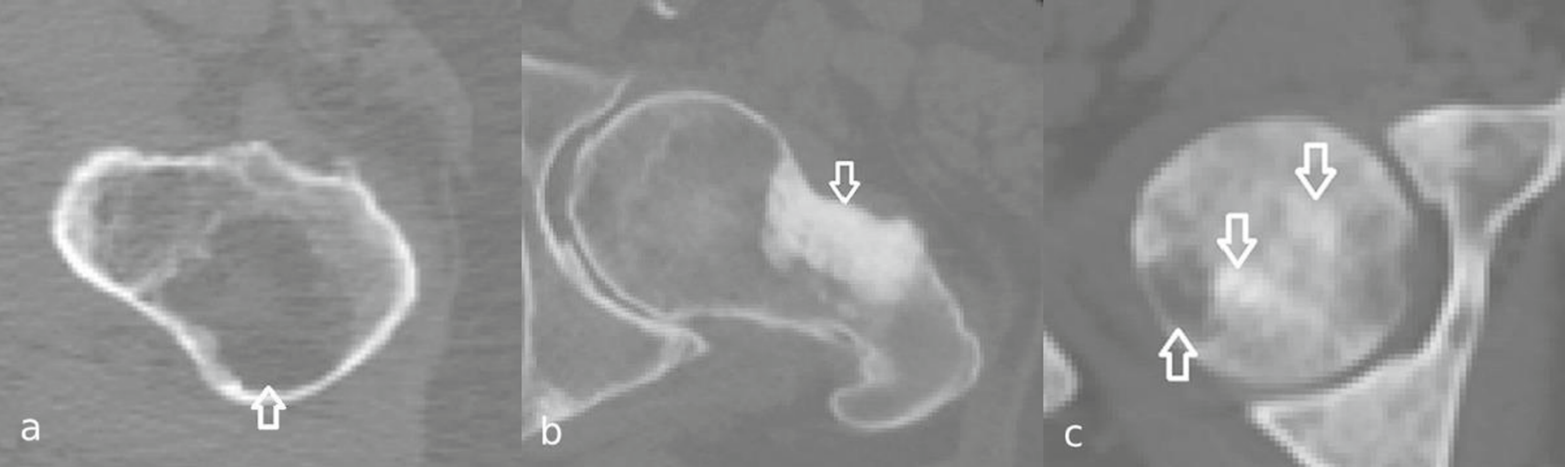
Arrows indicate femoral osteolytic **a**, osteoblastic **b**, and mixed **c** bone metastases in axial view

Bone is one of the most invaded tissues by the metastases of breast, prostate, lung, kidney, and thyroid cancer [1]. Approximately half of the primary tumors of these cancers metastasize to bone, with the femur, hip, spine, and skull as the most frequent sites [2]. Bone metastases can have three different types of appearance: osteolytic, osteoblastic, and mixed (Fig. 1). In osteolytic and osteoblastic metastases, bone absorption and bone formation are increased, respectively. Newly formed bone of osteoblastic metastases is known to be immature and of poor quality [3]. Bone metastases are often painful and may increase the risk of pathological fracture [4]. These pathological fractures result in increased rate of morbidity and mortality [5].

Patients with bone metastases are treated based on fracture risk: Patients with a high fracture risk are considered for prophylactic surgery to prevent pathological fracture, whereas patients with a low fracture risk are treated with conservative treatment such as radiotherapy for pain management. Higher-dose radiotherapy in order to induce remineralization is an option if the patient is not eligible for surgery [6].

Biomechanical finite element (FE) models can be used to evaluate metastatic bone fracture risk [7, 8]. In a prospective cohort study of our group, quantitative computed tomography (CT)-based FE models were created and bone strengths of femurs with predominantly osteolytic metastatic lesions were calculated to predict whether or not a fracture would occur [7, 8]. FE predictions had a higher sensitivity compared to those from clinical assessments. However, currently, the FE models are not yet applicable to femurs affected with osteoblastic metastases. To determine the mechanical properties within the FE model, calibrated bone densities from CT scans are obtained and since osteoblastic metastases appear very dense on CT scans this results in strong mechanical properties of the metastatic lesions although they are weaker in reality. A potential solution is to assign more appropriate material properties to the osteoblastic lesions to better simulate the mechanical behavior of the weakened tissue. For this purpose, exact segmentation of the metastatic lesions is important.

Additionally, segmentation of the metastatic lesions is important for radiotherapy planning. For this purpose, bone metastases are currently usually identified during visual inspection on CT scans and are segmented manually to determine exact radiation fields. Studies suggest that it is difficult to indicate exact edges of bone metastases, mainly in case of diffuse lesions [9–11]. Furthermore, studies investigated the inter-observer agreement of manual segmentation of bone metastases in the pelvis [12, 13], femoral head, and spine [12], as well as the scapula, humeral head, and ribs [13]. Their segmentations were done by a radiation oncologist, and relatively moderate agreement rates (0.46 and 0.61, respectively) were reported indicating that manual segmentation is challenging. However, the accuracy and repeatability of segmentation of femoral osteolytic, osteoblastic, and mixed bone metastases has not been studied before. Therefore, the aim of our study was to assess the inter- and intra-operator reliability of manual segmentation of femoral metastatic lesions. This was assessed for osteolytic, osteoblastic, and mixed lesions.

## Methods

To address the study aim, we used CT scans of patients with metastatic lesions which were segmented by two operators. Subsequently, the inter- and intra-operator reliability of the segmentations was assessed.

### CT scan

From our existing patient cohort (Approved by Commissie Mensgebonden Onderzoek regio Arnhem-Nijmegen. Reference number 2013/305) [8], we included 54 CT scans of femurs with bone metastases (19 osteolytic, 17 osteoblastic, and 18 mixed femurs) originating from multiple types of cancer such as prostate, breast, kidney, lung, esophagus, and plasma cell cancer (Table 3 appendix). All patients gave informed consent. CT scans were obtained from four radiotherapy institutes (Radboud University Medical Center, Leiden University Medical Center, Radiotherapeutic Institute Friesland Leeuwarden, and Bernard Verbeeten Institute Tilburg). As these images came from a multicenter clinical study, different voxel sizes were found (voxel size ranged from 0.86 × 0.86x2.5 mm^3^ to 1.27 × 1.27x3 mm^3^). Therefore, all 3-D lesion dimensions were displayed in cm^3^.

### Lesion segmentation

**Fig. 2:**
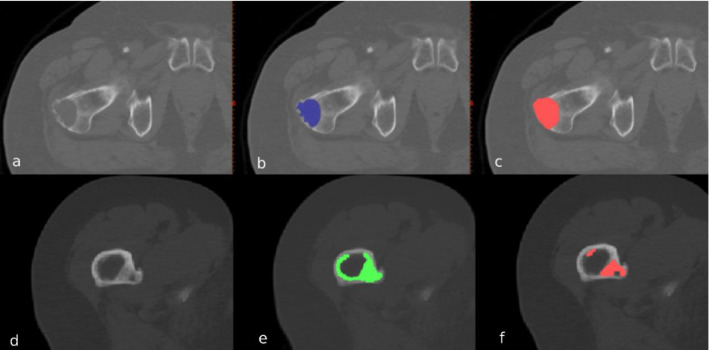
Example of an osteolytic (upper half) and osteoblastic (lower half) lesion in the proximal femur, and the manual segmentations by operators I (**b**, **e**) and II (**c**, **f**) in axial view. Cortical voxels of the osteolytic lesion were included by operator II (**c**), but not by operator I (**b**). The operators did not agree on the number of osteoblastic lesions: operator I only segmented one lesion (**e**), whereas operator II segmented two separate lesions (**f**)

Two operators with background in biomedical engineering (operator I) and biomedical sciences (operator II), who were familiar with segmentation, were trained by an experienced radiologist to identify and segment metastatic lesions. Prior to the start of the segmentation, operators practiced lesion segmentation on a trial metastatic lesion dataset for approximately 15 h followed by a feedback session with the experienced radiologist. Subsequently, they segmented all lesions of the 54 femurs twice with a four-week time interval, using Mimics 14.0 and 20.0 (Materialise, Leuven, Belgium (Fig. 2)).

Observers segmented the lesions, i.e., the voxels around the bone cortex or the boundary of the lesion itself were selected more accurately than the larger areas that are selected when lesions are segmented by a radiologist or a radiation oncologist. During segmentation, the following rules were used. First, we always searched for abnormalities in the bone relative to proximal and distal slices and to the contralateral femur. Second, lesions with altered cancellous bone were included in the segmentation. Third, missing bone in osteolytic lesions, especially in the diaphysis, was segmented. For this, we again compared the normal thickness of the bone relative to slices proximal and distal to the segmented slice and to the contralateral femur. Fourth, the sclerotic rim of osteolytic lesions was excluded from the segmentation. In case of mixed lesions, the sclerotic rim was included when it was very difficult to differentiate between osteolytic and osteoblastic metastases. Finally, for osteoblastic lesions and osteoblastic parts of mixed lesions, the segmentations were not limited to the normal boundary of the femoral bone, thus if the bone metastases grew out of the normal boundaries it was included in the segmentation.

### Inter- and intra-operator reliability

Segmentation similarities between and within operators were assessed using 3-D Dice coefficient (DC, formula 1). A and B represent two segmentation volumes:

1 $$ {\text{dice~coefficient}} = \frac{{2{\text{~}}\left| {{\text{A~}} \cap {\text{B}}} \right|}}{{{\text{~}}\left| {\text{A}} \right|{\text{~}} + \left| {\text{B}} \right|}} $$

The DCs were calculated for each femur, including all lesions within that femur. This was done, because operators did not necessarily agree on the number of lesions within one femur, causing some cases in which one operator segmented multiple smaller lesions, whereas the other operator segmented that as one larger lesion. We defined a DC˃0.7 as a good reliability, in accordance with a statistical image segmentation study [14]. Since our data were not distributed normally, Kruskal–Wallis one-way analysis of variance was used to test the mean DC differences between osteolytic (*n* = 19), osteoblastic (*n* = 17), and mixed (*n* = 18) lesions. *P*‐values below 0.05 were considered statistically significant. The results of mean DC analysis are reported as mean ± standard deviation (SD).

In addition, segmentation differences, i.e., the non-overlapping segmentation volume between and within operators, were calculated (formula 2, A and B represent two segmentation volumes).

2 $$ {\text{non}} - {\text{overlapping~segmentation~volume}} = {\text{~}}\left| {\text{A}} \right|{\text{~}} + \left| {\text{B}} \right| - 2{\text{~}}\left| {{\text{A}} \cap {\text{B}}} \right| $$

### Lesions size

To investigate whether the DC varies with the lesion size, mean segmentation volume of the first and second time (inter-operator) and operators I and II (intra-operator) was calculated per femur. A threshold of 60 cm^3^ was chosen, based on the observation that DC above this threshold was generally larger than 0.7, below and beyond which we compared the DCs. The results of mean segmentation volume are reported as mean ± standard deviation (SD).

## Results

### Data analyses

Only one out of four segmentation sets in this study (inter-operator I, inter-operator II, intra-operator I, and intra-operator II) resulted in good mean DC (˃0.7). Inter-operator DCs were 0.54 (± 0.28) and 0.50 (± 0.32) for the first and second segmentations, respectively, whereas intra-operator DCs were 0.56 (± 0.28) for operator I and 0.71 (± 0.23) for operator II. Table 1 summarizes the mean inter- and intra-operator DCs per lesion type and size. There was no statistically significant difference in the first and second segmentation mean DCs between osteolytic, osteoblastic, and mixed lesions (*p* = 0.86 and *p* = 0.41, respectively).

**Table 1:** Mean (± SD) inter- and intra-operator DCs and segmentation volume in cm^3^ per lesion type

		Inter-operator		Intra-operator	
		First segmentation	Second segmentation	Operator I	Operator II
Mean DC (± SD)	Osteolytic	0.54 (± 0.26)	0.45 (± 0.31)	0.56 (± 0.26)	0.63 (± 0.27)
	Osteoblastic	0.57 (± 0.28)	0.60 (± 0.27)	0.60 (± 0.28)	0.78 (± 0.12)
	Mixed	0.51 (± 0.29)	0.46 (± 0.34)	0.52 (± 0.3)	0.74 (± 0.23)
Mean segmentation volume (± SD)	Osteolytic	14.48 (± 13.83)	12.88 (± 10.79)	14.44 (± 13.65)	12.92 (± 11.76)
	Osteoblastic	33.35 (± 32.42)	37.12 (± 40.56)	32.51 (± 36.74)	37.95 (± 37.77)
	Mixed	41.2 (± 27.47)	35 (± 28.39)	28.72 (± 24.78)	47.48 (± 35.82)

**Fig. 3:**
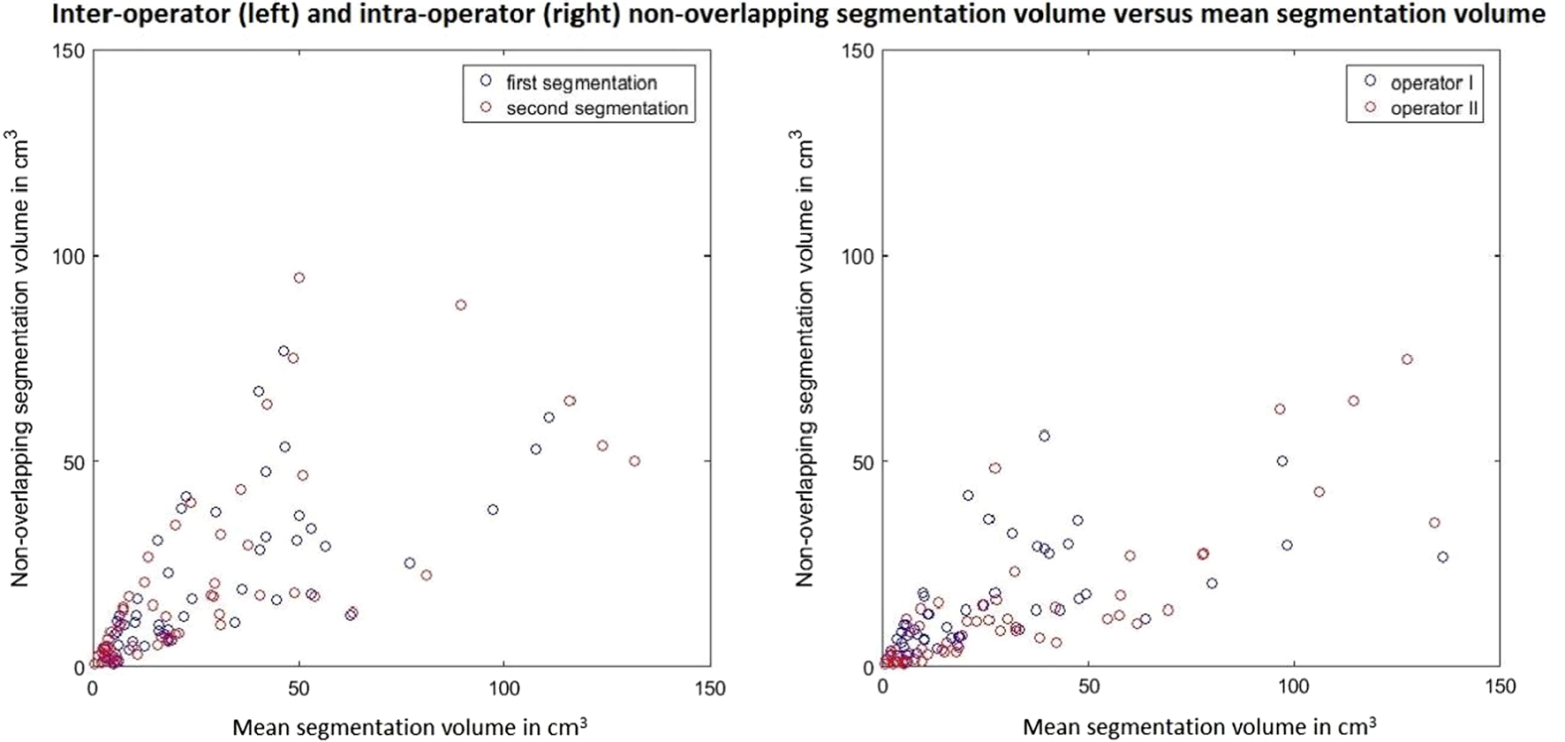
Inter- and intra-operator non-overlapping segmentation volume versus mean segmentation volume. Each dot represents all segmented lesions of one femur. In the inter-operator figure (left), the blue dots represent the first segmentations and the red dots the second segmentations. In the intra-operator figure (right), the blue dots represent the segmentations by operator I and the red dots represent the segmentations by operator II

The mean inter-operator non-overlapping segmentation volumes of the first and second segmentations were 22.66 (± 24.22) and 20.69 (± 22.47) cm^3^, respectively, whereas the mean intra-operator non-overlapping segmentation volumes were 14.53 (± 12.74) and 13.94 (± 16.51) cm^3^ for operators I and II, respectively. The non-overlapping segmentation volume increased with increasing mean segmentation volume (Fig. 3).

### Lesion size

**Fig. 4:**
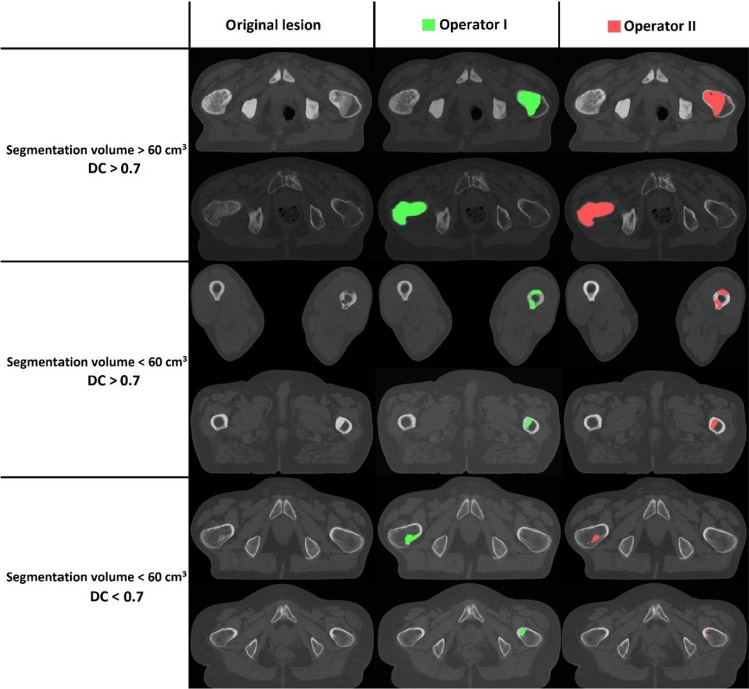
Examples of segmentation comparisons between operators I and II for different lesion volumes and DCs above or below 0.7. Each row shows the axial view of a metastatic lesion either in the proximal part or in the diaphysis

**Fig. 5:**
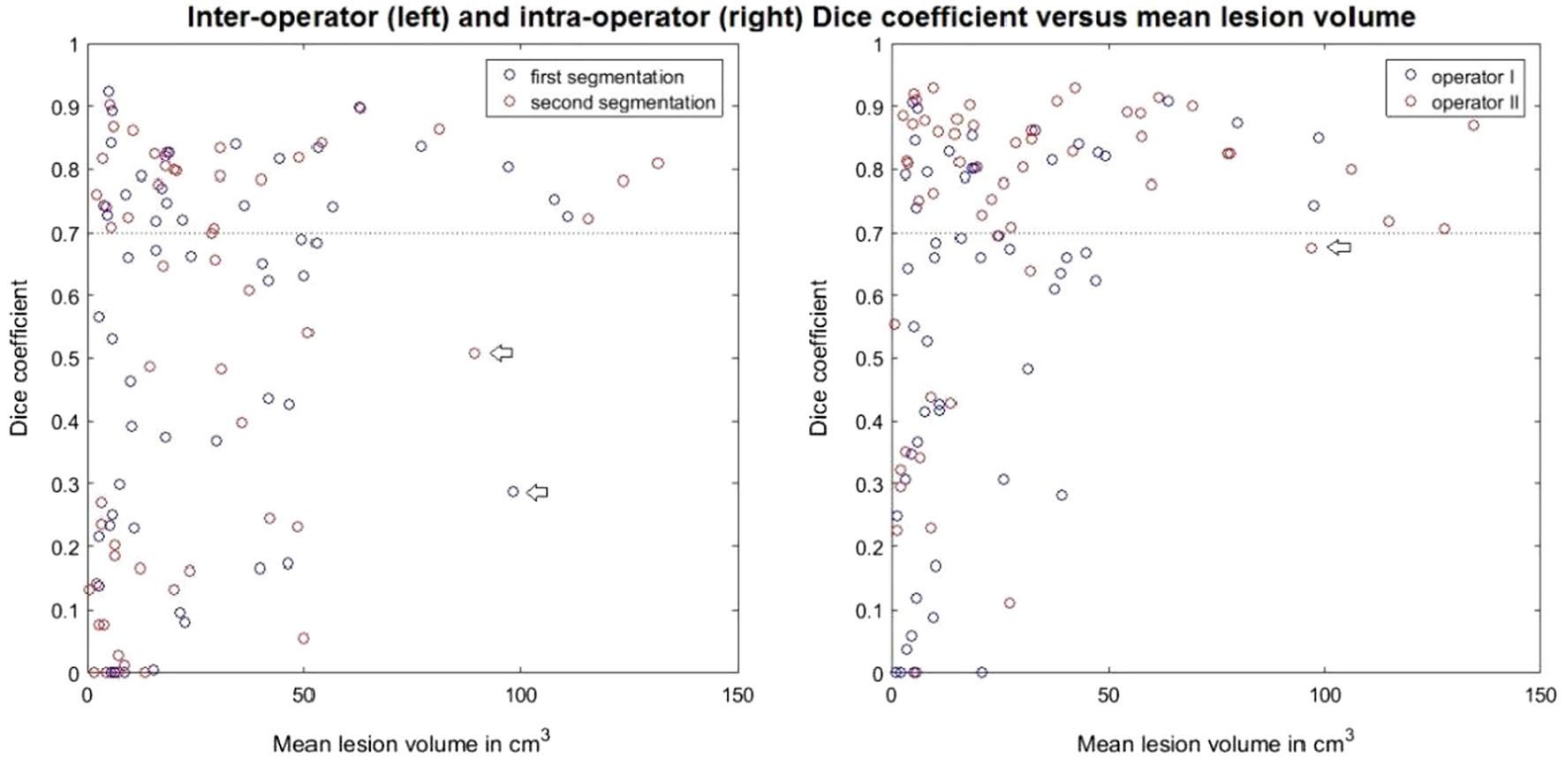
Inter- and intra-operator DC versus mean segmentation volume. Note that in the inter-operator figure (left) mean segmentation volume indicates the mean over segmentation volume of operators I and II. The blue dots represent the first measurements and the red dots represent the second measurements. The arrows indicate two femurs which scored DCs below 0.7, although they are in the group of larger lesions (˃60 cm^3^). In the intra-operator figure (right), mean segmentation volume indicates the mean over segmentation volumes of the first and second segmentations of each individual operator. The blue dots represent operator I and the red dots represent operator II. The arrow indicates a femur that scored DC below 0.7, although it was in the group of larger lesions (˃60 cm^3^)

For operator I, the mean segmentation volumes were 22.75 (± 28.39) and 22.03 (± 27.66) cm^3^ for the first and second segmentations, respectively, whereas these were 30.91 (± 32.24) and 33.74 (± 37.32) cm^3^ for the first and second segmentations of operator II, respectively. In general, the inter- and intra-operator DCs were higher for femurs with larger lesion segmentation volumes (Fig. 4). Accordingly, for femurs with larger lesion segmentation volumes (˃60 cm^3^), mean inter- and intra-operator DCs were good (> 0.7), whereas for femurs with smaller lesion segmentation volumes, the DCs were lower (Table 2). Of the femurs with larger mean segmentation volumes, 83% and 93% were segmented with good inter- and intra-operator DCs (> 0.7), respectively, whereas only 39% and 50% of the femurs with smaller mean segmentation volumes scored good inter- and intra-operator DCs, respectively (Fig. 5).

**Table 2:** Mean inter- and intra-operator DCs per lesion size

		Inter-operator	Intra-operator
Mean DC (± SD)	˃60 cm^3^	0.74 (± 0.16)	0.81 (± 0.07)
	˂60 cm^3^	0.49 (± 0.3)	0.61 (± 0.28)

## Discussion

In the current study, we aimed to evaluate the inter- and intra-operator reliability of manual segmentation of osteolytic, osteoblastic, and mixed femoral metastatic lesions. For this purpose, metastatic lesions of 54 femurs were segmented twice by two operators. Since in most cases it was difficult to detect the bone metastases, only one out of four segmentation sets (inter-operator I, inter-operator II, intra-operator I, and intra-operator II) resulted in good mean DC (˃0.7). There was no statistically significant difference in the mean inter- and intra-operator DCs between osteolytic, osteoblastic, and mixed lesions.

The results of the segmentations showed that despite the same learning experience and instructions, segmentation remains a subjective procedure. The two operators in our study, who had no clinical experience, were trained by an expert radiologist. However, in our opinion they were adequately trained for this specific task and there are no indications that better segmentation agreements are achieved if they are done by domain experts [15]. Hence, the lesions in this study were not segmented by an experienced radiologist. Additionally, when demonstrating a subset consisting of four segmentations of the same femora to the clinical experts, they were deemed adequate. This also proves the extreme difficulty of segmenting these types of lesions in a reproducible and robust manner, also for radiologists with clinical experience.

The results also showed that both non-overlapping segmentation volume and DC varied with the size of the lesion. Non-overlapping segmentation volume increased with an increase in lesion size. The results also convincingly showed that a high percentage (83% and 93%) of the larger lesions scored good inter- and intra-operator DC.

For most of the lesions, the segmentation volume differences originated from unclear boundaries which made it difficult for the operators to select the correct metastatic voxels during their visual inspections. In agreement with our findings, moderate mean agreement of 0.46 (range 0.15–0.75) and 0.61 (range 0.31–0.81) was reported by Gerlich et al. [12] and Raman et al. [13], respectively. Moreover, a study by Hoyte et al. [16] reported that the manual segmentation of the female pelvis floor organs was adequate for locating the organs but poor for segmenting structural boundaries. This is one of the most important points in differentiating between the ability of humans to recognize abnormal patterns and automated computer-assisted systems.

Similar studies in the past reported different reliability rates of manual segmentations of tissue abnormalities or whole target organs. For instance, Fiez et al. [17] reported a good agreement between their observers investigating the inter- and intra-operator reliability on segmentation of brain damage lesions, while Tingelhoff et al. [18] studied the replicability of the manual segmentation of sinuses and concluded that this is a time-consuming procedure which is not replicable. Accordingly, they suggested using automatic segmentation approaches.

This study has some limitations. The first limitation concerns our outcome measure. In contrast with the studies that use boundary-based measurements such as the Hausdorff distance to investigate the segmentation similarity, we used the volume-based Sorensen-Dice coefficient as an outcome measure since our operators did not necessarily agree on the number of lesions within a femur (Fig. 2).

As a second limitation, it should be noted that we obtained four lesion segmentation sets for each of the 54 femurs, and relatively large differences were typically found, for which it was impossible to know which segmentation was closest to quantify the true lesion in the bone. Hence, there was no ground truth. The absence of a ground truth could be addressed by utilizing cadaver bones in which lesions are drilled simulating osteolytic lesions. The empty holes will show maximal contrast and it is expected to have the maximum inter- and intra-operator reliability as there would be a clear CT intensity difference of the lesion with respect to the non-lesion surrounding tissue. However, the translation to true clinical lesions would be difficult to make as in reality there will be a mixture of osteolytic and osteoblastic lesions. For this reason, such a cadaver assessment was not implemented in this study.

## Conclusion

In conclusion, manual segmentation of femoral bone metastases is very challenging and resulted in unsatisfactory mean reliability values (DC between 0.4 and 0.7). Hence, the development of a segmentation protocol to reduce the inter- and intra-operator segmentation variation as the first step and the development and use of a computer-assisted segmentation method as the second step are warranted. Automatic segmentation methods based on deep learning algorithms may assist clinicians in their attempts to adequately segment lesions in the metastasized femurs and technicians to calculate bone strength of femurs with osteoblastic lesions.

**Table 3:** Information of all 54 patients included in the study

Patient ID	Sex (*m* = male; *f* = female)	Age (years)	Primary cancer type	Radiotherapy treatment
PT-01	m	55	Prostate	1 × 8 Gy
PT-02	m	59	Kidney	2 × 8 Gy
PT-03	m	82	Lung	5 × 4 Gy
PT-04	m	74	Prostate	1 × 8 Gy
PT-05	m	68	Multiple myeloma	1 × 8 Gy
PT-06	f	61	Lung	1 × 8 Gy
PT-07	f	50	Breast	1 × 8 Gy
PT-08	m	73	Prostate	1 × 8 Gy
PT-09	m	74	Non-Hodgkin lymphoma	10 × 3 Gy
PT-10	f	66	Breast	1 × 8 Gy
PT-11	f	60	Lung	After surgery 5 × 4 Gy
PT-12	m	70	Prostate	5 × 4 Gy
PT-13	f	68	Breast	1 × 8 Gy
PT-14	m	65	Prostate	1 × 8 Gy
PT-15	m	72	Prostate	1 × 8 Gy
PT-16	m	87	Prostate	1 × 8 Gy
PT-17	m	75	Prostate	1 × 8 Gy
PT-18	m	72	Prostate	1 × 8 Gy
PT-19	m	75	Prostate	5 × 4 Gy
PT-20	m	95	Prostate	No RT
PT-21	f	41	Breast	1 × 8 Gy
PT-22	f	67	Lung	1 × 8 Gy
PT-23	f	74	Multiple myeloma	1 × 8 Gy
PT-24	f	55	Kidney	5 × 4 Gy
PT-25	m	60	Multiple myeloma	Not available
PT-26	m	80	Prostate	1 × 8 Gy
PT-27	m	70	Prostate	5 × 4 Gy
PT-28	m	80	Prostate	1 × 8 Gy
PT-29	m	82	Prostate	1 × 8 Gy
PT-30	f	72	Lung	1 × 8 Gy
PT-31	m	87	Prostate	Not available
PT-32	f	61	Breast	1 × 8 Gy
PT-33	f	69	Multiple myeloma	5 × 4 Gy
PT-34	m	53	Lung	1 × 8 Gy
PT-35	m	66	Prostate	1 × 8 Gy
PT-36	m	77	Multiple myeloma	5 × 4 Gy
PT-37	m	48	Lung	1 × 8 Gy
PT-38	m	55	Prostate	1 × 8 Gy
PT-39	f	51	Lung	1 × 8 Gy
PT-40	f	65	Lung	1 × 8 Gy
PT-41	f	67	Lung	1 × 8 Gy
PT-42	m	86	Prostate	1 × 8 Gy
PT-43	f	68	Breast	1 × 8 Gy
PT-44	m	57	Lung	1 × 8 Gy
PT-45	f	78	Lung	5 × 4 Gy
PT-46	m	82	Prostate	1 × 8 Gy
PT-47	m	64	Esophagus	1 × 8 Gy
PT-48	f	68	Breast	1 × 8 Gy
PT-49	f	75	Breast	1 × 8 Gy
PT-50	m	66	Prostate	1 × 8 Gy
PT-51	f	69	Breast	1 × 8 Gy
PT-52	m	57	Multiple myeloma	5 × 4 Gy
PT-53	m	77	Lung	1 × 8 Gy
PT-54	f	58	Breast	1 × 8 Gy

### Supplementary Information

Below is the link to the electronic supplementary material.

Supplementary file1 (XLSX 54 kb)

## Data Availability

The data used and/or analyzed during the current study are available from the corresponding author on reasonable request.

## Appendix

See Table 3.
